# Investigating the Effects of Tissue-Specific Extracellular Matrix on the Adipogenic and Osteogenic Differentiation of Human Adipose-Derived Stromal Cells Within Composite Hydrogel Scaffolds

**DOI:** 10.3389/fbioe.2019.00402

**Published:** 2019-12-11

**Authors:** Arthi Shridhar, Brian G. Amsden, Elizabeth R. Gillies, Lauren E. Flynn

**Affiliations:** ^1^Department of Chemical and Biochemical Engineering, Thompson Engineering Building, The University of Western Ontario, London, ON, Canada; ^2^Bone and Joint Institute, The University of Western Ontario, London, ON, Canada; ^3^Department of Chemical Engineering, Queen's University, Kingston, ON, Canada; ^4^Department of Chemistry, The University of Western Ontario, London, ON, Canada; ^5^Department of Anatomy and Cell Biology, Schulich School of Medicine and Dentistry, The University of Western Ontario, London, ON, Canada

**Keywords:** adipose-derived stromal cells, cell culture models, decellularization, extracellular matrix (ECM), hydrogel composites, methacrylated chondroitin sulfate, lineage-specific differentiation

## Abstract

While it has been postulated that tissue-specific bioscaffolds derived from the extracellular matrix (ECM) can direct stem cell differentiation, systematic comparisons of multiple ECM sources are needed to more fully assess the benefits of incorporating tissue-specific ECM in stem cell culture and delivery platforms. To probe the effects of ECM sourced from decellularized adipose tissue (DAT) or decellularized trabecular bone (DTB) on the adipogenic and osteogenic differentiation of human adipose-derived stem/stromal cells (ASCs), a novel detergent-free decellularization protocol was developed for bovine trabecular bone that complemented our established detergent-free decellularization protocol for human adipose tissue and did not require specialized equipment or prolonged incubation times. Immunohistochemical and biochemical characterization revealed enhanced sulphated glycosaminoglycan content in the DTB, while the DAT contained higher levels of collagen IV, collagen VI and laminin. To generate platforms with similar structural and biomechanical properties to enable assessment of the compositional effects of the ECM on ASC differentiation, micronized DAT and DTB were encapsulated with human ASCs within methacrylated chondroitin sulfate (MCS) hydrogels through UV-initiated crosslinking. High ASC viability (>90%) was observed over 14 days in culture. Adipogenic differentiation was enhanced in the MCS+DAT composites relative to the MCS+DTB composites and MCS controls after 14 days of culture in adipogenic medium. Osteogenic differentiation studies revealed a peak in alkaline phosphatase (ALP) enzyme activity at 7 days in the MCS+DTB group cultured in osteogenic medium, suggesting that the DTB had bioactive effects on osteogenic protein expression. Overall, the current study suggests that tissue-specific ECM sourced from DAT or DTB can act synergistically with soluble differentiation factors to enhance the lineage-specific differentiation of human ASCs within 3-D hydrogel systems.

## Introduction

The extracellular matrix (ECM) provides a complex microenvironment that has tissue-specific structural, biochemical, and biomechanical properties, which can direct cell function (Scott, 1995). Recognizing the biological importance of the ECM, there is growing emphasis on the design of cell culture platforms that integrate compositional elements of the native cellular milieu (He et al., 2008; Kasten et al., 2014; Beringer et al., 2016). For example, hydrogel systems have been developed that incorporate ECM-derived proteins (e.g., collagen, fibronectin), adhesive peptide sequences [e.g., arginine-glycine-aspartic acid (RGD) motifs], and/or growth factors in an effort to enhance bioactivity and tune the response of encapsulated cell populations (He et al., 2008; Kasten et al., 2014; Beringer et al., 2016).

Decellularization is a useful approach for generating bioscaffolds with a complex ECM composition that can mimic the native cellular microenvironment within tissues (Hoshiba et al., 2010). There is increasing interest in applying tissue-specific ECM in biomaterials-based strategies to direct the lineage-specific differentiation of stem or progenitor cell populations both *in vitro* and *in vivo* (Stern et al., 2009; DeQuach et al., 2011; French et al., 2012). While tissue-specific compositional effects are often postulated, to date many studies have focused on characterizing responses to single ECM sources (Brown et al., 2015; Beck et al., 2016) or rely on comparisons drawn to control groups such as collagen gels or tissue culture plastic that differ in multiple properties that can affect the cellular response (French et al., 2012; Yu et al., 2013). Hence, there is a need to develop 3-D platforms that enable the systematic comparison of the compositional effects of decellularized tissues to be able to more fully assess the mechanisms involved and potential benefits of applying tissue-specific ECM in cell-instructive culture and delivery systems.

Hydrogels can be designed to enable cell encapsulation with high viability and offer great versatility for the development of customizable 3-D cell culture models (Nicodemus and Bryant, 2008). While ECM-derived hydrogels have been synthesized from pepsin-digested decellularized tissues, these hydrogels tend to be mechanically weak and show low stability unless chemically crosslinked (Turner and Flynn, 2012; Visser et al., 2015). Further, proteolytic digestion alters the structure and composition of the ECM, which may affect its bioactivity (Beachley et al., 2015; Williams et al., 2015). Recognizing these limitations, hydrogel composites incorporating micronized ECM as a cell-instructive component represent a promising alternative (Cheung et al., 2014; Almeida et al., 2016; Beachley et al., 2018). Applying a composite approach can combine the benefits of hydrogel systems with the innate bioactivity of the ECM. Parameters such as the hydrogel phase, ECM source and particle size, cell type(s) and seeding density can be altered to tune the desired cellular response (Cheung et al., 2014; Brown et al., 2015; Hayami et al., 2015; Shridhar et al., 2017). For example, composites developed in our lab incorporating 5 wt% human decellularized adipose tissue (DAT) particles within methacrylated glycol chitosan (MGC) and methacrylated chondroitin sulfate (MCS) hydrogels (Cheung et al., 2014) were shown to promote the adipogenic differentiation of encapsulated human adipose-derived stem/stromal cells (ASCs) when cultured in adipogenic medium, with enhanced viability and adipogenesis in the MCS-based composites (Cheung et al., 2014). While not the focus of our study, modifications to the number of crosslinkable moieties within the hydrogel phase could be used to adjust scaffold properties such as stiffness (Bryant et al., 2004; Ondeck and Engler, 2016) and degradation (Bryant et al., 2004; Ornell et al., 2019), which can also modulate the cellular response.

Building from this, the current study extends our models to generate tissue-specific hydrogel composites incorporating micronized decellularized trabecular bone (DTB). Due to variability in the reported osteogenic activity of commercially-available demineralized bone matrix (DBM) (Peterson et al., 2004) and the widespread use of detergents for bone decellularization (Gardin et al., 2015; Lee et al., 2016), initial work focused on the development and validation of a new detergent-free method to obtain DTB from bovine tissues. Detergent-free decellularization protocols are advantageous for preserving ECM components that may be readily extracted with detergents, and avoid potential cytotoxicity concerns associated with residual detergents that can be challenging to remove at the end of processing (Cebotari et al., 2010). The protocol was designed to complement our patented detergent-free decellularization process for human adipose tissue (Flynn, 2010), and avoid the requirements for mechanical milling prior to processing, specialized equipment, and prolonged incubation times in strong acids (e.g., HCl) used in published bone decellularization protocols.

Applying our composite hydrogel cell encapsulation strategy previously established with the DAT, we fabricated composites incorporating a higher concentration (8 wt%) of micronized DAT or DTB within MCS hydrogels. The MCS was selected as it provides a supportive environment for human ASCs (Cheung et al., 2014; Brown et al., 2015), and because chondroitin sulfate is a native ECM component within both adipose tissue and bone (Boskey and Posner, 1984; Zwick et al., 2018). These platforms were then applied to investigate the effects of the tissue-specific ECM on the viability and lineage-specific differentiation of encapsulated human ASCs. While we have previously shown that a variety of DAT-based scaffolds have pro-adipogenic effects on human ASCs (Flynn, 2010; Yu et al., 2013, 2017; Cheung et al., 2014; Han et al., 2015), this is the first study to systematically compare the cellular response within structurally and biomechanically similar platforms generated with another decellularized tissue source, as well as the first investigation of osteogenic differentiation within our hydrogel systems. To test the hypothesis that the ECM composition has tissue-specific effects in directing the lineage-specific differentiation of the encapsulated human ASCs, adipogenesis and osteogenesis were characterized *in vitro* within MCS+DAT, MCS+DTB, and MCS hydrogels under differentiation and proliferation media conditions to probe the “conductive” and “inductive” effects of the ECM sources, respectively.

## Methods

### Materials

Unless otherwise specified, all chemicals and reagents were purchased from Sigma Aldrich and used as received. All studies were performed within a biohazard level II certified lab facility with institutional approval from Western University (Permit # BIO-UWO-0293).

### Bovine Bone Procurement and Decellularization

Bovine trabecular bone was sourced from the vertebrae in the tail region of fresh food-grade tissues collected from the abattoir in Mount Brydges, ON, Canada. The trabecular bone was extracted, cut into pieces (~2 × 2 × 2 mm) and subjected to a new 4-day detergent-free decellularization protocol, which was developed based on methods used for producing commercially-available DBM (Pacaccio and Stern, 2005), as well as methods for adipose tissue decellularization established in our lab (Flynn, 2010). The process was performed at a reagent:starting tissue ratio of 25 mL/g. First, 3 freeze-thaw cycles were performed between −80 to 37°C in hypotonic solution [10 mM Tris, 5 mM EDTA, 1% antibiotic/antimycotic (ABAM) and 0.014 mM phenylmethylsulfonylfluoride (PMSF)], with replacement of the solution between each cycle and agitation at 200 rpm on an orbital shaker during thawing. Next, the tissue was demineralized at room temperature in 23% formic acid (StatLab, Texas) for 8 h under agitation at 300 rpm. The processed bone was then incubated in absolute isopropanol supplemented with 1% ABAM and 0.014 mM PMSF under agitation at 200 rpm and 37°C overnight to extract lipids. Following this, the tissue was rinsed in Sorensen's phosphate buffer (SPB) rinse solution [8 g/L NaCl, 200 mg/L KCl, 1 g/L Na_2_HPO_4_, and 200 mg/L KH_2_PO_4_ (pH 8.0)], supplemented with 1% ABAM, three times for 30 min each at room temperature and then incubated in SPB digest solution (55 mM Na_2_HPO_4_, 17 mM KH_2_PO_4_, 4.9 mM MgSO_4_•7H_2_O) supplemented with 12.5 mg RNase Type III (from bovine pancreas), 15,000 U DNase Type II (from bovine pancreas), 2,000 U Lipase type VI-S (from porcine pancreas) and 1% ABAM overnight at 37°C under agitation at 200 rpm. Finally, the tissue was rinsed in the SPB rinse solution three times for 30 min each at room temperature, followed by two 30 min rinses in deionized water (dH2O) and freezing at −80°C and lyophilization.

### Validation of the Bone Decellularization Protocol

To confirm the efficacy of the decellularization protocol at extracting nuclear content and to qualitatively assess collagen distribution relative to native bone, samples of DTB (*n* = 3 samples, *N* = 3 tissue donors) were paraffin-sectioned and stained with 4′,6-diamidino-2-phenylindole (DAPI) and Masson's trichrome using previously published methods (Flynn, 2010; Yu et al., 2017). Native tissue control samples were demineralized in formic acid overnight prior to sectioning and staining. For quantitative assessment of decellularization, double stranded DNA (dsDNA) was extracted using the DNeasy Blood and Tissue Kit (Qiagen, Germany) and quantified using the PicoGreen® dsDNA quantification assay (Life technologies) according to the manufacturers' protocols. The resulting data was normalized to the initial dry mass of the processed tissues.

### Adipose Tissue Procurement, Decellularization and ASC Isolation

Subcutaneous adipose tissue was collected with informed consent from patients undergoing elective lipo-reduction surgeries at the University Hospital and St. Joseph's Hospital in London, ON, Canada with Human Research Ethics Board approval from Western University (HREB# 105426). The tissue was either decellularized to obtain DAT (Flynn, 2010) or used for ASC isolation within 2 h of procurement (Flynn et al., 2006) following previously-published methods. The summary of cell donor information for all *in vitro* studies can be found in Supplementary Table S1.

### Comparison of the ECM Composition in DTB and DAT

The collagen distribution in the DTB and DAT samples (*n* = 3 samples, *N* = 2 tissue donors) was qualitatively probed through Picrosirius red staining using established methods (Junqueira et al., 1979). Slides were visualized using a Nikon Optiphot-pol polarizing microscope (Nikon, Tokyo) under circularly polarized light and images were captured with an Infinity 2-3 CCD camera (Lumenera, Ottawa, Canada) at 10X magnification.

The hydroxyproline and dimethylmethylene (DMMB) blue assays were performed to quantitatively measure the total collagen and sulphated GAG content, respectively, in the DTB and DAT samples (*n* = 3 samples, *N* = 3 tissue donors pooled prior to the trial) based on published methods (Morissette Martin et al., 2019). The resulting data was normalized to the initial dry mass of the processed tissues. To prepare cryo-milled tissue samples for the assays, two 10 mm milling balls and minced ECM were loaded into a 25 mL cryo-milling chamber (Retsch, Germany) and submerged in liquid nitrogen for 3 min. The ECM was then cryo-milled for 3 min at a frequency of 30 Hz using the Mixer Mill MM 400 milling system (Retsch, Germany). Prior to performing the assays, the cryo-milled ECM was digested with 60 mU/mL proteinase K (Promega, Madison, USA) in Tris-EDTA (200 nM Tris-HCl, 200 nM EDTA, pH 7.5) overnight under agitation at 1,200 rpm and 65°C, followed by a 10 min incubation at 95°C to inactivate the enzyme.

To further compare the ECM composition, DTB and DAT samples (*n* = 3 samples, *N* = 2 tissue donors) were processed, cryo-sectioned and stained for collagen type I (dilution 1:100, ab34710, Abcam), collagen type IV (dilution 1:100, ab6586, Abcam), collagen type V (dilution 1:300, ab7046, Abcam), collagen type VI (dilution 1:300, ab6588, Abcam), laminin (dilution 1:150, ab23750, Abcam) and fibronectin (dilution 1:200, ab11575, Abcam) according to previously published methods (Morissette Martin et al., 2019). Positive tissue controls (Supplementary Figure S1) and controls with no added primary antibody were included during all experiments. Images were acquired with a Zeiss Imager M2 microscope (Zeiss Canada, Toronto, ON, Canada) from 4 to 5 fields of view on each sample. Semi-quantitative analysis of the relative expression levels was performed using ImageJ and results were reported as a percentage of the positive signal area normalized to the total tissue area.

### DTB and DAT Cryo-Milling, Characterization, and Staining

Bovine DTB (pooled from 3 donors) and human DAT (pooled from 5 donors) were cryo-milled as described in the previous section and sieved using stainless steel sieves (W.S. Tyler, St. Catharines, ON, Canada) to select for particles between 45 and 125 μm. The choice of size was based on previous studies indicating that smaller particles within MCS+DAT hydrogels were favorable for adipogenesis by promoting cell-cell and cell-ECM interactions (Brown et al., 2015). The particle size distributions of the DAT and DTB particles were analyzed using a Malvern Mastersizer 2000 (Malvern Instruments Ltd., Worcestershire, United Kingdom) using previously published protocols (Brown et al., 2015) and a Kolmogorov–Smirnov test with *p* < 0.05 was performed to assess whether the particle size distributions for the DAT and DTB particles were statistically equivalent.

To enable visualization of the ECM particles within the hydrogels using confocal microscopy, the DAT and DTB particles were pre-labeled with an amine reactive AlexaFluor® 405 carboxylic acid succinimidyl ester (Life technologies) based on previously published methods (Yu et al., 2017). Prior to encapsulation, the ECM particles were decontaminated in 70% ethanol overnight, followed by 3 successive 1 h rinses in phosphate buffered saline (PBS).

### Methacrylation of Chondroitin Sulfate

Methacrylated chondroitin sulfate (MCS) was synthesized using established procedures with chondroitin sulfate (50 kg/mol, LKT Technologies, St. Louis) and methacrylate anhydride (Shridhar et al., 2017) to obtain a final degree of substitution of 20%, as confirmed by ^1^H NMR spectroscopy with a Varian Inova 400 spectrometer (Varian, USA) (Supplementary Figure S2).

### Composite Hydrogel Synthesis and Characterization

Composite MCS hydrogels incorporating cryo-milled DAT or DTB, along with MCS controls, were synthesized for characterization of crosslinking efficiency and mechanical properties. Cryo-milled ECM [8% (w/v)] and Irgacure 2959 photoinitiator [final concentration: 0.05% (w/v)] were combined with the MCS pre-polymer solution consisting of MCS dissolved in water (for gel content studies) or PBS (for mechanical testing) to obtain a final MCS concentration of 10% (w/v). The resulting pre-gel solution was transferred to a cylindrical mold (1 mL syringe; Diameter: 4.78 mm) and crosslinked for 4 min (2 min on one side, followed by 2 min on the other) using long-wavelength ultraviolet light (~365 nm) at an intensity of 12 mW/cm^2^. The hydrogels were cut into 50 μL gels and used for subsequent studies.

The gel content was assessed to confirm that the crosslinking efficiency was similar between the groups (*n* = 3 samples per group/trial, *N* = 3 trials) following previously-published protocols (Cheung et al., 2014). Mechanical testing was also performed to compare the bulk compressive properties of the MCS+DAT, MCS+DTB, and MCS groups. Hydrogels (*N* = 5) were crosslinked and swollen to equilibrium in PBS (pH 7.4) and maintained at 37°C prior to testing. The diameter of the hydrogels was measured using calipers immediately before testing. Unconfined compression measurements were conducted using a CellScale UniVert system (Waterloo, Canada) equipped with a 0.5 N load cell in a PBS bath maintained at 37°C. Samples were initially put through 2 cycles of preconditioning to a maximum of 10% strain at a rate of 0.05%/s (Yahia and Drouin, 1990). Cyclic compression testing was then performed with a 0.01 N preload applied at every cycle and a total strain of 10% at a rate of 0.05%/s, for a total of 4 cycles (Antoine et al., 2014). The Young's modulus (E) was obtained from the slope of the linear region of the nominal stress-strain curve using the stress at 4 and 10% strain values as boundary conditions.

### Photo-Encapsulation of hASCs Within MCS Hydrogels

*In vitro* culture studies were performed to assess ASC viability and differentiation toward the adipogenic and osteogenic lineages following encapsulation within the MCS+DAT, MCS+DTB, and MCS hydrogels. For these studies, passage 3 human ASCs were suspended in proliferation medium comprised of DMEM:Ham's F-12 medium supplemented with 10% fetal bovine serum (FBS; Thermo Scientific Hyclone, Cat. # SH30396) and 100 U/mL penicillin and 0.1 mg/mL streptomycin (1% pen-strep). The ASCs were then combined with the MCS pre-gel solution prepared in PBS (20:80 ratio), to obtain a final concentration of 10 x 10^6^ ASCs/mL of pre-gel, followed by photo-cross-linking as described in the previous section. The hydrogels were cut into 50 μL gels, each containing ~500,000 cells, and cultured in 12-well inserts (Greiner Bio-one, Germany).

### Cell Viability and Cellularity Analysis of Encapsulated ASCs

For the cell viability studies, samples were cultured in adipogenic differentiation medium, osteogenic differentiation medium, and proliferation medium as a control. Differentiation was induced after 24 h in culture in proliferation medium, following rinsing with PBS. In all cases, fresh media was provided to all samples every 2–3 days. The adipogenic differentiation medium was composed of DMEM:Ham's F12 supplemented with 33 μM biotin, 17 μM pantothenate, 10 μg/mL transferrin, 100 nM hydrocortisone, 66 nM human insulin, 1 nM triiodothyronine, and 1% pen-strep, with 0.25 mM isobutylmethylxanthine (IBMX) and 1 μg/mL troglitazone supplemented for the first 3 days (Flynn, 2010). The osteogenic medium was composed of DMEM-low glucose (Gibco, Cat # 11885084) supplemented with 10% FBS, 150 μM ascorbate-2-phosphate, 10 mM β-glycerophosphate, 10 nM dexamethasone, 10 nM 1,25-dihydroxyvitamin D3, and 1% pen-strep (de Girolamo et al., 2007).

Cell number and viability within the hydrogels were assessed by staining using the LIVE/DEAD® Viability/Cytotoxicity Kit for mammalian cells (Life Technologies Inc., Burlington, ON) at 7 and 14 d post-induction of differentiation (*n* = 3 samples per group/trial, *N* = 3 trials with different ASC donors) following previously published methods (Cheung et al., 2014), with visualization using a Zeiss Multiphoton LSM 510 META confocal microscope.

### Characterization of Adipogenic Differentiation Within the Composite Hydrogels

*In vitro* studies were performed to probe the effects of incorporating the tissue-specific ECM within the MCS hydrogels on the adipogenic differentiation of encapsulated ASCs at 14-days post-induction in adipogenic differentiation medium. Hydrogels maintained in proliferation medium were included as non-induced (NI) controls. As additional controls for the GPDH assay, 12-well tissue culture polystyrene (TCP) plates were seeded with P3 ASCs at a density of 50,000 cells/cm^2^ and cultured in both media formulations.

To quantitatively assess differentiation, intracellular GPDH enzyme activity was measured using a GPDH Enzyme Activity Measurement Kit (Kamiya Biomedical Corporation, Cat. # KT-010, Seattle, WA, USA) (*n* = 3 samples per group/trial, *N* = 3 trials with different ASC donors) as per previously-published methods (Cheung et al., 2014; Brown et al., 2015). For each donor, the data was normalized to the non-induced TCP group for comparative purposes. In addition, Bodipy® 493/503 staining (Thermo Scientific) was performed to visualize intracellular lipid accumulation in the hydrogels (*n* = 3 samples per group/trial, *N* = 3 trials with different ASC donors) following the manufacturer's protocol. The hydrogels were imaged using a Zeiss Multiphoton LSM 510 META confocal microscope, with images obtained at 25X magnification.

### Characterization of Osteogenic Differentiation

Similar to the adipogenic differentiation study, osteogenic differentiation was probed in the MCS+DAT, MCS+DTB, and MCS hydrogels cultured in osteogenic medium or proliferation medium as a non-induced control. For the ALP assay, P3 ASCs seeded at a density of 5,000 cells/cm^2^ on laminin-coated (Sigma, Cat. # L2020; 1.6 μg/cm^2^) TCP and cultured in both media formulations were included as additional controls.

ALP enzyme activity was measured at 7 and 14 d post-induction of differentiation (*n* = 3 samples per group/trial, *N* = 3 trials with different ASC donors), following published methods (Yu et al., 2017). For each cell donor, the data was normalized to the TCP group at 7 d under non-induced conditions for comparative purposes. In addition, matrix mineralization was qualitatively assessed in the hydrogel groups at 28 d post-induction using the OsteoImage^TM^ (Lonza, Germany) kit (*n* = 3 samples per group/trial, *N* = 2 trials with different ASC donors) as per the manufacturer's protocol. As a negative control, cell-free hydrogels without ASCs were also prepared, cultured, and imaged using a Zeiss Multiphoton LSM 510 META confocal microscope at 25X magnification.

### Statistical Analysis

All numerical data are expressed as mean ± standard deviation (SD). Unless otherwise stated, all statistical analyses were performed using GraphPad Prism 6 (GraphPad Software, San Diego, CA) by one-way or two-way ANOVA with a Tukey's *post-hoc* comparison of the means. Differences were considered statistically significant at *p* < 0.05 unless otherwise noted.

## Results

### The Decellularization Protocol Effectively Extracted Cells From Bovine Trabecular Bone

**Figure 1:**
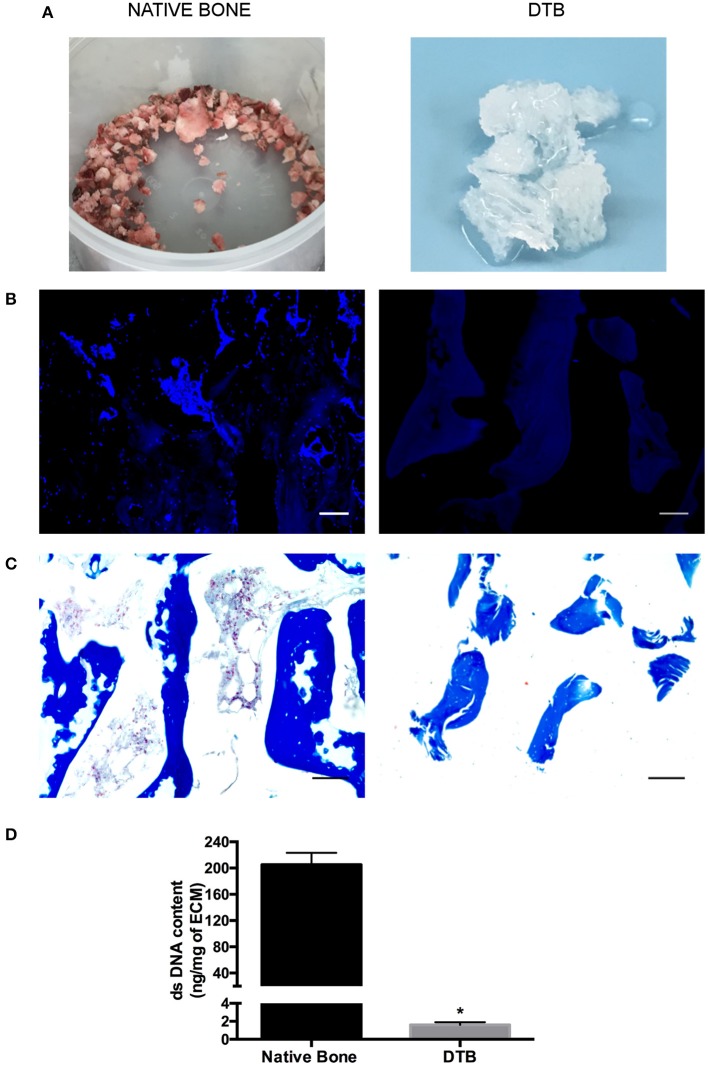
Confirmation that the new detergent-free decellularization protocol effectively extracted the cellular content from the bovine trabecular bone. **(A)** Macroscopic evaluation of native and decellularized bone samples suggested the effective removal of bone marrow constituents based on the uniform white appearance of the DTB. **(B)** DAPI and **(C)** Masson's trichrome staining showed no detectable nuclei within the bone marrow compartment, or in the lacunae within the collagenous region of the bone. Scale bar: 100 μm. **(D)** PicoGreen® quantification of double stranded DNA content (per mg dry weight) in decellularized samples showed a significant reduction in the dsDNA content compared to native tissues (*p < 0.05) (*n* = 3, *N* = 3).

At the end of processing, the DTB samples consistently had a uniform white appearance, suggestive of the successful removal of cells including bone marrow constituents (Figure 1A). Similarly, DAPI (Figure 1B) and Masson's Trichrome (Figure 1C) staining confirmed that no nuclei were visualized in either the bone marrow compartment or within the trabeculae, supporting the efficacy of the approach. No qualitative differences were observed in the collagen staining patterns between the pre- and post-decellularization samples. The quantitative assessment of dsDNA content (Figure 1D) confirmed effective decellularization, with a >100-fold decrease in dsDNA content in the DTB in comparison with the native tissues.

### The ECM of DTB and DAT Are Compositionally Distinct

**Figure 2:**
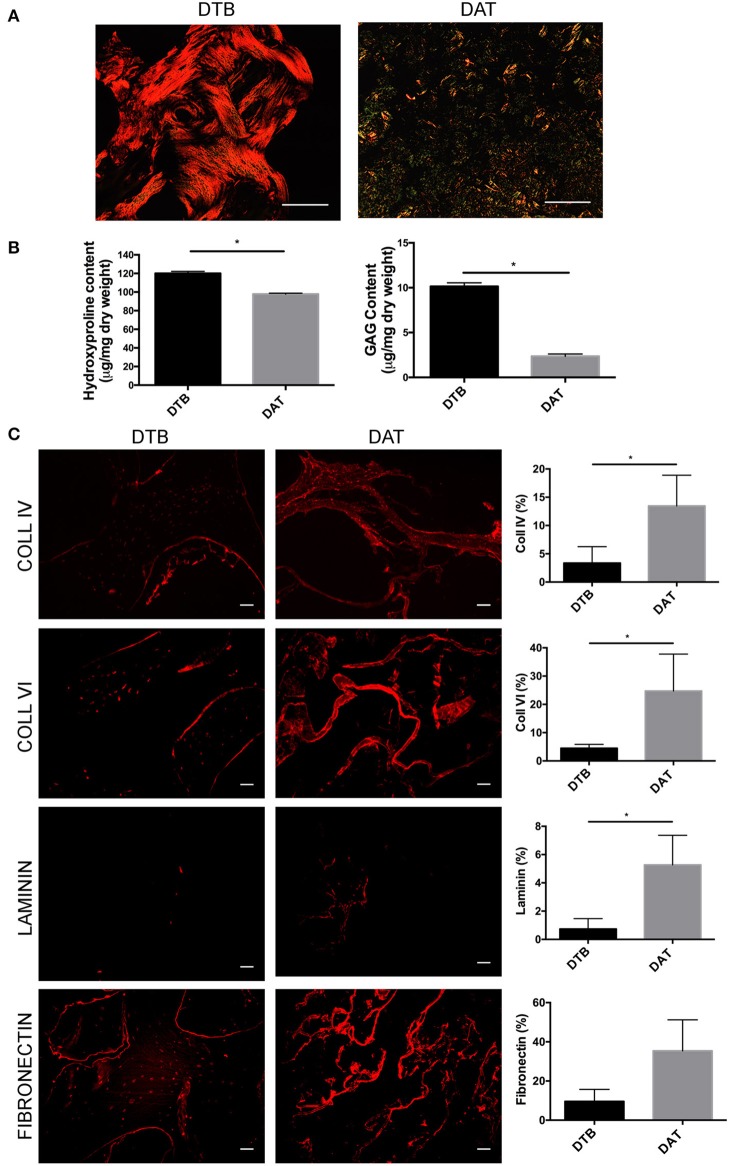
The DTB has a distinct ECM structure and composition as compared to DAT. **(A)** Picrosirius red staining and the resulting birefringence visualized under a polarized light microscope indicated predominantly thicker fibrillar collagen types in the DTB, and thinner fibers in the DAT. **(B)** Higher levels of collagen and sulphated GAG content were measured in the DTB as compared to the DAT (*p < 0.05, *n* = 3, *N* = 3 pooled). **(C)** Immunohistochemical staining and semi-quantitative comparative analyses of the DTB and DAT displayed significantly higher amounts of collagen IV, VI, and laminin in the DAT. Fibronectin was qualitatively more abundant in the DAT, with regional variability leading to no significant difference between the groups (*n* = 3, *N* = 2 tissue donors, 4–5 images per sample) (*p < 0.05) Scale bar: 100 μm.

Picrosirius red staining (Figure 2A) was used to compare the distribution and organization of collagens in the DTB and DAT. The collagen in the DTB was arranged in a dense woven pattern that predominantly appeared red/orange, indicative of thicker fibrillar collagen types. In contrast, the DAT was arranged in a less dense network that included a blend of thick and thin fibers, as seen by the green/yellow birefringence patterns. Quantitative analysis of total collagen content through the hydroxyproline assay showed that the DTB contained a significantly higher amount of hydroxyproline on a dry mass basis as compared to the DAT (Figure 2B). Further, the DMMB assay indicated that the sulphated GAG content was ~5-fold higher in the DTB as compared to the DAT (Figure 2B).

Immunohistochemical staining was performed to further probe the differences in the distribution and abundance of key ECM constituents in the DTB and DAT (Figure 2C). Collagen type IV and VI were localized at the periphery of the collagenous regions of the trabeculae and around the lacunae in the DTB, while they were more uniformly distributed throughout the DAT. Semi-quantitative image analysis indicated that both ECM sources contained similar relative amounts of collagen type I and collagen type V (Supplementary Figure S3). Notably, the DAT contained ~4-fold higher collagen type IV, ~6-fold higher collagen type VI, and ~5-fold higher laminin expression as compared to the DTB (Figure 2C). In general, both tissue sources displayed low expression of laminin, with substantial regional variability in its distribution. Although fibronectin appeared to be qualitatively more abundant in the DAT, there was regional variability that resulted in the levels not being significantly different from the DTB (Figure 2C).

### The MCS Composite Hydrogels Provided a Controlled Platform for 3-D Cell Culture

**Figure 3:**
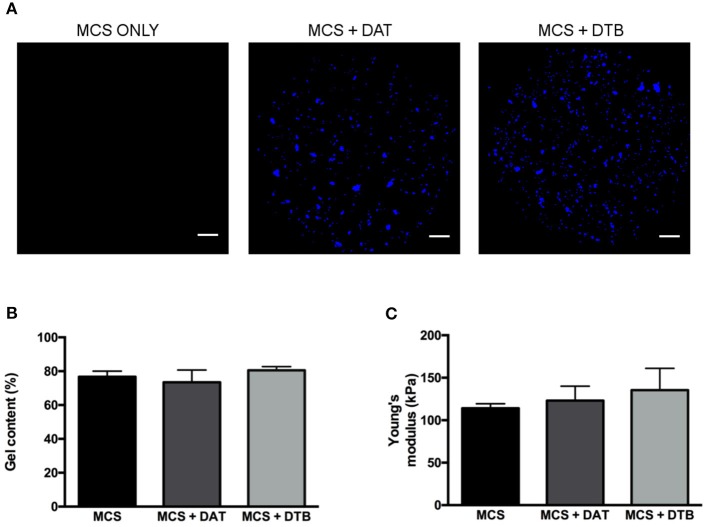
Confirmation that the MCS, MCS+DAT and MCS+DTB hydrogel systems exhibited similar bulk properties. **(A)** Confocal image analysis showing that the ECM particles (blue) were well distributed throughout the hydrogel phase. Scale bars: 1 mm. No significant differences were observed between the three hydrogel systems in terms of **(B)** gel content (*n* = 3 samples per group/trial, *N* = 3 trials) and **(C)** Young's moduli, measured under unconfined compression in PBS at 37°C (*N* = 5).

Initial testing focused on confirming that the DTB and DAT particles could be incorporated at a high concentration (8 wt%) within the MCS hydrogels to generate stable constructs with similar crosslinking efficiencies and bulk compressive properties so that they could be applied as controlled 3-D platforms for exploring the effects of the ECM composition on ASC differentiation. Prior to encapsulation, particle size analyses confirmed that the size distributions of the DAT and DTB particles were statistically equivalent, eliminating size as a potential confounding variable (Supplementary Figure S4). Based on visual inspection, the ECM particles were homogenously distributed throughout the hydrogel phase (Figure 3A) and the platforms remained stable over the maximum period (28 days) of cell culture. The gel content, an indicator of crosslinking efficiency, was statistically equivalent for the different hydrogel groups (Figure 3B), suggesting that the ECM particles did not interfere with the cross-linking process. Further, there were no significant differences in the measured hydrogel bulk compressive moduli, with values of 114 ± 5 kPa for the MCS group, 123 ± 17 kPa for the MCS+DAT group, and 135 ± 26 kPa for the MCS+DTB group (Figure 3A).

### The MCS Composite Hydrogels Provided a Cell-Supportive Platform for Long-Term Culture Studies

**Figure 4:**
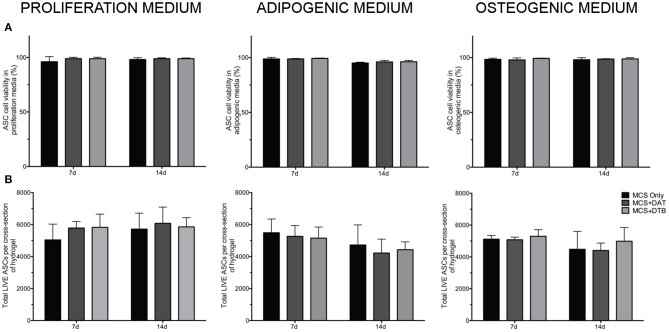
High cell viability and similar cell densities within the hydrogels were confirmed under all media conditions tested. **(A)** Cell viability of ASCs encapsulated within MCS, MCS+DAT, MCS+DTB hydrogels under proliferation, adipogenic, and osteogenic media conditions maintained >90% cell viability over the 14 d culture period. **(B)** Analysis of total live cell number indicated no significant differences between any of the groups under all media conditions, eliminating cell density as a factor mediating differentiation (*n* = 3 samples per group/trial, *N* = 3 trials with different ASC donors).

LIVE/DEAD® staining with confocal image analysis showed high cell viability (>90%) for all of the hydrogel groups and culture media conditions at both 7 and 14 days, indicating that the hydrogels provided a cell-supportive microenvironment for the human ASCs (Figure 4A). Further, there were no significant differences in the total number of live cells per x-y plane between the groups for any of the media conditions at either time point (Figure 4B). Representative confocal images of LIVE/DEAD®-stained hydrogel groups at 7 d are shown in Supplementary Figure S5.

### Incorporating the DAT Enhanced the Adipogenic Differentiation of the Human ASCs Encapsulated Within the Hydrogel Composites

**Figure 5:**
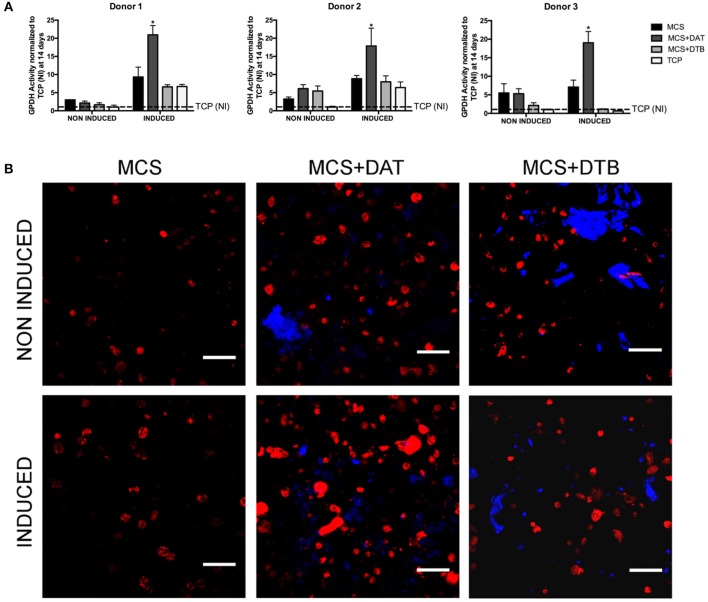
ASC adipogenesis was enhanced in the MCS+DAT hydrogel composites. **(A)** GPDH enzyme activity was significantly enhanced in the MCS+DAT hydrogels cultured in adipogenic medium (induced) for 14 days as compared to all other groups for all three ASC donors studied (*p < 0.05). Data is normalized to the non-induced (NI) TCP samples at 14 days. (*n* = 3 samples per group/trial, *N* = 3 trials with different ASC donors). **(B)** BODIPY^©^ staining (red) of lipid droplets in encapsulated ASCs at 14 d post-induction showed qualitatively enhanced intracellular lipid accumulation in the MCS+DAT composites cultured in adipogenic (induced) and proliferation (non-induced) medium, with the induced cells showing a more mature phenotype with larger lipid droplets. The labeled ECM particles within the composites appear blue. Scale bar: 100 μm.

Under the induced conditions, significantly higher GPDH enzyme activity was observed at 14 days in the MCS+DAT hydrogels relative to all other groups for all three ASC donors studied (Figure 5A). No differences were observed between the groups for the non-induced samples maintained in proliferation medium, suggesting that there was a synergistic effect of the tissue-specific ECM in combination with the soluble differentiation factors present in the adipogenic medium. The qualitative assessment of intracellular lipid accumulation (Figure 5B) indicated that there were a higher number of differentiating cells in the MCS+DAT group as compared to the MCS+DTB and MCS groups under both induced and non-induced conditions. Notably, a qualitatively higher response with larger lipid droplets consistent with a more mature adipocyte phenotype was observed in the MCS+DAT samples cultured in adipogenic medium. These results again support the synergistic effects of the tissue-specific ECM and the soluble differentiation factors in the media, although the qualitatively enhanced intracellular lipid accumulation in the MCS+DAT group relative to the MCS+DTB and MCS groups cultured in proliferation media does suggest that the adipose-derived ECM had some adipo-inductive capacity.

### Incorporating the DTB Modulated Osteogenic Marker Expression in the Human ASCs Encapsulated Within the Hydrogel Composites

**Figure 6:**
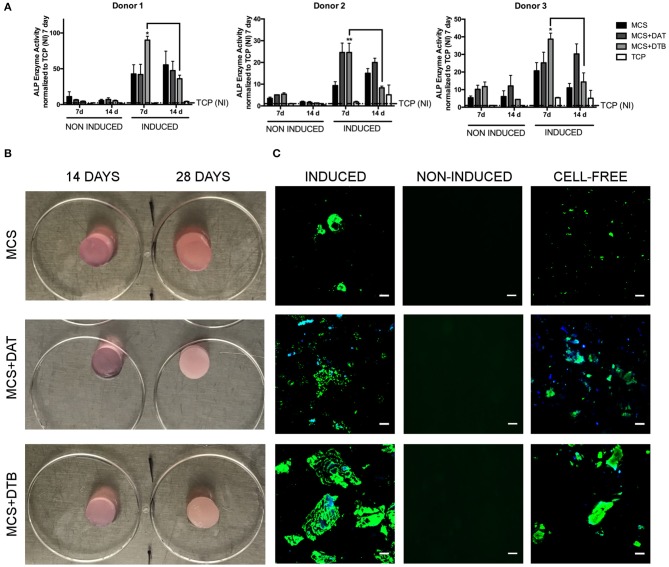
ASC expression of osteogenic markers was modulated by the incorporation of DTB within the hydrogel composites. **(A)** A peak in ALP enzyme activity at 7 d was observed in the MCS+DTB group cultured in osteogenic differentiation medium for all three ASC donors, suggesting that the DTB modulated osteogenic protein expression. Data is normalized to the non-induced (NI) TCP samples at 7 days. (*,**p < 0.05) (*n* = 3 samples per group/trial, *N* = 3 trials with different ASC donors) * statistically different than all other groups at 7 days, ** statistically different than all other groups except MCS+DAT at 7 days. Bars indicate a significant difference between 7 and 14 days (*p* < 0.05). **(B)** Macroscopic images of the MCS, MCS+DAT and MCS+DTB hydrogels at 14 and 28 days post-induction showing an increasingly opaque appearance in the hydrogels cultured in osteogenic medium, suggestive of matrix mineralization. **(C)** Positively-stained regions showing matrix mineralization (green) were observed at 28 days in the hydrogels cultured in osteogenic medium (induced), with qualitatively greater matrix mineralization around the ECM particles (blue) in the MCS+DTB composites. No staining was detected in the samples cultured in proliferation medium (non-induced controls). However, positive staining for matrix mineralization was observed in the cell-free hydrogel controls. Scale bar: 50 μm.

For all three cell donors, ALP enzyme activity was enhanced at 7 days in the ASCs encapsulated within the hydrogels relative to the TCP controls under both induced and non-induced conditions, indicating that culturing within the 3-D platforms modulated ASC osteogenic protein expression (Figure 6A). In general, ALP enzyme activity levels were higher in the induced hydrogel samples cultured in osteogenic differentiation medium as compared to their corresponding non-induced controls, supporting that the combination of the ECM and the soluble factors present in the osteogenic medium induced a more potent response. In fact, in assessing the response in the samples cultured in proliferation medium, no notable osteo-inductive effects were observed in any of the scaffold groups over the time frame of the current study. However, significantly higher ALP activity was observed in the MCS+DTB group cultured in osteogenic medium at 7 days (compared to all other groups for cell donors 1 & 3 and compared to all other groups except MCS+DAT for cell donor 2), followed by a significant decline in activity from 7 to 14 d for all three ASC donors. These findings suggest that the DTB may have had bioactive effects on the progression of osteogenic differentiation when combined with soluble factors in the osteogenic medium.

Over 28 d, the hydrogel constructs became increasingly opaque and macroscopically more rigid, suggestive of matrix mineralization (Figure 6B). OsteoImage^TM^ stained hydrogels showed positive staining in all three groups when cultured under osteogenic medium conditions, with qualitatively enhanced mineral deposition around the ECM particles in the MCS+DTB group (Figure 6C). The lack of detectable positive staining for mineralization in the non-induced samples maintained in proliferation medium indicated that there was no residual mineral content in the DTB that was contributing to the positive signal in that group. Consistent with the ALP findings, these results also support that the DTB alone was not sufficient to induce mineralized matrix production by the encapsulated ASCs, but that there were synergistic effects of the tissue-specific ECM with the soluble osteogenic differentiation factors in the media. However, marked positive staining was observed in the ASC-free control hydrogels cultured in osteogenic medium for 28 days, indicating that there was non-physiological calcium phosphate precipitation induced by the culture conditions.

## Discussion

Recognizing that there is an inverse relationship between adipogenesis and osteogenesis (Chen et al., 2016a), in the current study we expanded our hydrogel composite platform to explore the effects of tissue-specific ECM on the adipogenic and osteogenic differentiation of encapsulated human ASCs. As a first step in this study, a straightforward decellularization method was developed for bovine trabecular bone that avoided the use of potentially cytotoxic detergents, strong acids and specialized equipment (e.g., isostatic pressurization machines) and complemented our established detergent-free adipose decellularization approach. Our new protocol reproducibly extracted the cellular content from both within the trabeculae and the bone marrow compartment. While it is acknowledged that the bovine sourcing of the bone may have impacted the cellular response, bovine bone is physiochemically similar to human bone (Figueiredo et al., 2010), and ECM macromolecules are generally well-conserved across species (Scott, 1995).

Applying the DAT and DTB, we successfully established conditions for generating stable composites containing higher concentrations of ECM as compared to our previous studies (8 vs. 5 wt%), while retaining similar crosslinking efficiencies and bulk mechanical properties to pure MCS controls, providing a controlled basis for the comparative studies. The ECM particle size was also controlled, based on previous findings that the DAT particle size can influence ASC adipogenesis (Brown et al., 2015). Of note, while the bulk mechanical properties were similar between the hydrogel groups, it is recognized that cells in contact with the ECM particles may have experienced different mechanical properties at the cellular level, which may have influenced the cellular response. Another potential limitation is that cryomilling the decellularized tissues alters the ECM structure, creating a more homogenous mixture of particles as compared to the heterogeneous regional distribution of the ECM within native stem cell niches, which provides complex spatially-organized cues that play important roles in directing cell fate (Cortiella et al., 2010; Nakayama et al., 2013). However, there is a growing body of evidence to support that the ECM composition retained within scaffolds derived from processed decellularized tissues, including hydrogels, foams, and particles, can be harnessed to help direct stem cell differentiation. The ability to source ECM from bulk tissues is attractive in terms of scale-up and translation, although it may be interesting in future studies to explore whether bioactivity could be modulated by decellularizing more selective regions of certain tissues, such as separating the basement membrane-rich region of the adipose-derived ECM from the dense fibrous collagens that likely play a more structural role within the tissues (Flynn, 2010).

All three hydrogel systems provided a highly supportive microenvironment for the encapsulated ASCs. Cell density is an important mediator of both adipogenesis and osteogenesis, with high cell densities shown to promote adipogenesis and low cell densities shown to promote osteogenesis in both 2-D and 3-D systems (Bitar et al., 2008; Xue et al., 2013; Brown et al., 2015). The similar live cell densities between the three groups under all media formulations tested eliminated cell density as a possible confounding factor that may have impacted the cellular response.

The current study clearly demonstrated that adipogenesis was enhanced in the ASCs encapsulated in the MCS+DAT composites cultured in adipogenic differentiation medium, suggesting there was a synergistic effect of the tissue-specific ECM with the soluble factors present in the medium. The pro-adipogenic effects were specific to the DAT, with similar and lower levels of adipogenesis observed in the MCS+DTB composites and MCS controls. The higher levels of collagen type IV, collagen type VI and laminin in the DAT may have had instructive effects in mediating adipogenic differentiation, as the basement membrane is known to play a fundamental role in adipogenesis (Kawaguchi et al., 1999; Uriel et al., 2008; Hsueh et al., 2017). Further supporting this possibility, chitosan and alginate composite hydrogels incorporating purified laminin or laminin-derived sequences have successfully enhanced the adipogenic differentiation of pre-adipocytes and ASCs *in vitro* (Chen et al., 2015, 2016b; Hsueh et al., 2017).

Interestingly, all of the platforms supported intracellular lipid accumulation under proliferation medium conditions containing serum, known to suppress adipogenic differentiation in ASCs (Park et al., 2013), with the MCS+DAT group demonstrating qualitatively the most consistent and uniform distribution of maturing adipocytes. Similar findings were observed in our previous study of MCS and MCS+DAT hydrogels (Brown et al., 2015), and may be related to the 3-D culture environment promoting a more rounded cellular morphology favorable for adipogenesis (McBeath et al., 2004), as no lipid accumulation was observed in the non-induced TCP controls. The qualitatively enhanced intracellular lipid staining in the non-induced MCS+DAT group suggests that the adipose-derived ECM had an adipo-inductive effect on the encapsulated ASCs, although there was no observed difference in the GPDH enzyme activity levels between the groups at 14 days. Future studies could focus on further probing these effects at later timepoints, including additional markers of adipogenic differentiation at both the gene and protein level, to develop a better understanding of the instructive capacity of the adipose-derived ECM.

Similar to our previous findings supporting the adipo-conductive and adipo-inductive effects of the DAT (Flynn, 2010; Yu et al., 2013, 2017; Cheung et al., 2014; Brown et al., 2015), a number of studies have highlighted that decellularized bone ECM can enhance mesenchymal stromal cell (MSC) osteogenesis under differentiation medium conditions (Ravindran et al., 2015; Hung et al., 2016; Paduano et al., 2017), and in the absence of soluble osteo-inductive factors (Hung et al., 2016; Paduano et al., 2017). Glycosaminoglycans (GAGs), abundantly expressed in bone tissue (Boskey and Posner, 1984), are known to modulate osteoblast attachment and mineralization, potentially through the action of GAG-sequestered growth factors (Slater et al., 2009). As such, the elevated levels of GAG content in the DTB, combined with the GAG-rich environment of the MCS, may be favorable for the osteogenic differentiation of the encapsulated ASCs.

Using alkaline phosphatase activity as a marker of bone formation (Boskey and Posner, 1984), the ASCs encapsulated within the MCS hydrogel platforms showed an enhanced response as compared to the TCP controls, suggesting that the 3-D culture systems may be advantageous for osteogenesis. Indeed, other types of 3-D hydrogels have been reported to promote osteogenic differentiation as compared to TCP (Nguyen et al., 2017), with GAG-based hydrogels promoting osteogenesis both *in vitro* and *in vivo* (Bae et al., 2014). The enhanced ALP activity levels at 7 days, followed by a decline at 14 days in the MCS+DTB composites cultured in osteogenic differentiation medium suggested that the DTB may have modulated the progression of differentiation in the presence of soluble osteogenic factors. During differentiation, ASCs go through an initial proliferative phase, followed by the synthesis and organization of collagen type I and finally, matrix mineralization (Boskey and Posner, 1984). ALP enzyme activity peaks during the initial stages, after which it declines before peaking again prior to matrix mineralization (Boskey and Posner, 1984). Since osteogenic markers can be upregulated at multiple stages throughout the differentiation process (Huang et al., 2007), a detailed time-course investigation including osteogenic markers at both the gene and protein level would be recommended for future studies. These studies may reveal more subtle osteo-inductive effects of the DTB not observed in the current study.

The qualitatively greater matrix mineralization around the DTB particles suggested a possible benefit of the tissue-specific ECM in enhancing mineralization when combined with soluble osteogenic factors. Mineralization was also observed around the DAT particles, consistent with collagen type I fibrils serving as nucleation sites for calcium phosphate deposition and crystallization (Boskey and Posner, 1984). However, the non-physiological matrix mineralization within the cell-free hydrogels cultured in osteogenic medium confounded the interpretation of the mineralization data. Notably, it has been reported that media supplementation with >2 mM β-glycerol phosphate (BGP) led to over 70% non-physiological precipitation of mineral within *in vitro* cell cultures (Chung et al., 1992). The high concentrations of phosphates included in standard osteogenic formulations in the form of ascorbate-2-phosphate (≥50 μM) and BGP (10 mM) is a concern (Ravindran et al., 2015; Hung et al., 2016; Paduano et al., 2017). Further, many studies have reported matrix mineralization without showing cell-free scaffold controls. Interestingly, it has been shown that the initiation and progression of mineralization work independently and the sustained presence of organic phosphates may not be required for bone nodule mineralization (Bellows et al., 1991). The alternative supplementation of phosphates through inorganic means (e.g., sodium dihydrogen phosphate) has also shown greater matrix mineralization despite use at lower concentrations (Schäck et al., 2013). Hence, these findings emphasize the broader need in the field to refine current osteogenic media formulations, and enforce the use of proper controls to be able to accurately interpret the differentiation response.

Overall, our study has provided evidence to support there is a synergistic effect of applying tissue-specific ECM in combination with soluble factors to modulate ASC differentiation within 3-D hydrogel composites, with the DAT having clear cell-instructive effects in mediating ASC adipogenesis. Notably, ASCs are known to be more predisposed toward the adipogenic lineage as compared to bone marrow-derived MSCs (Strioga et al., 2012), which may have contributed to the potent adipogenic response and responsiveness to the DAT. In contrast, bone marrow-derived MSCs have been reported to show enhanced osteogenic potential, as evaluated by gene expression data, ALP activity and matrix mineralization (Strioga et al., 2012). As such, in future studies it would be interesting to compare the adipogenic and osteogenic responses of ASCs vs. bone marrow-derived MSCs within the hydrogel composites under refined culture conditions, to explore the possibility of cell-type mediated effects.

## Conclusion

In the current study, a reproducible detergent-free decellularization method was developed for bovine trabecular bone that did not require excessive physical pre-processing, specialized equipment, strong acids, or prolonged incubation times. Hydrogel composites were fabricated that allowed for the stable encapsulation of a high density of cryo-milled ECM and human ASCs, with high ASC viability sustained over 14 days in culture. The platforms could be extended to include a range of ECM types and could function as injectable cell delivery systems. This strategy offers versatility as the hydrogel composition, ECM particle size and concentration, mechanical properties, and cell density could be tuned to enable screening of multiple parameters on the response of encapsulated cell populations. The platforms were designed to have similar structural and biomechanical properties, to facilitate assessment of the compositional effects of the ECM on ASC differentiation. Our findings indicated that the MCS+DAT composites provided a pro-adipogenic microenvironment for human ASCs, further supporting the rationale for applying a tissue-specific approach in designing cell culture and delivery systems for adipose regeneration. While the interpretation of the osteogenic response was more challenging, the ALP and matrix mineralization suggested that the DTB had bioactive effects and may have modulated ASC osteogenesis in the presence of soluble differentiation factors. Future studies should focus on refining the osteogenic media conditions to avoid the issues with non-physiological mineralization, likely due to the high phosphate concentrations in the formulation. Further, time course studies assessing additional markers at the gene and protein expression levels would be valuable for probing the conductive and inductive effects of the tissue-specific ECM. In addition, the models could be extended to include other ECM sources and regenerative cell types, such as bone marrow-derived MSCs or induced pluripotent cells (iPSCs), to assess the broader utility of the hydrogel composite platform and benefits of applying a tissue-specific approach.

## Data Availability Statement

The raw data supporting the conclusions of this manuscript will be made available by the authors, without undue reservation, to any qualified researcher.

## Ethics Statement

The studies involving human participants were reviewed and approved by the Human Research Ethics Board at The University of Western Ontario. The patients/participants provided their written informed consent to participate in this study.

## Author Contributions

AS, LF, and EG conceptualized and designed the study. AS performed the experimental studies and analyzed the data in consultation with LF, EG, and BA. AS and LF wrote the manuscript, with editorial feedback provided by EG and BA.

### Conflict of Interest

The authors declare that the research was conducted in the absence of any commercial or financial relationships that could be construed as a potential conflict of interest.

## References

[B1] Almeida H. V. Eswaramoorthy R. Cunniffe G. M. Buckley C. T. O'Brien F. J. Kelly D. J. (2016). Fibrin hydrogels functionalized with cartilage extracellular matrix and incorporating freshly isolated stromal cells as an injectable for cartilage regeneration. Acta Biomater. 36, 55–62. 10.1016/j.actbio.2016.03.008 26961807

[B2] Antoine E. E. Vlachos P. P. Rylander M. N. (2014). Review of collagen I hydrogels for bioengineered tissue microenvironments: characterization of mechanics, structure, and transport. Tissue Eng. Part B Rev. 20, 683–696. 10.1089/ten.teb.2014.0086 24923709 PMC4241868

[B3] Bae M. S. Ohe J. Y. Lee J. B. Heo D. N. Byun W. Bae H.. (2014). Photo-cured hyaluronic acid-based hydrogels containing growth and differentiation factor 5 (GDF-5) for bone tissue regeneration. Bone 59, 189–198. 10.1016/j.bone.2013.11.019 24291420

[B4] Beachley V. Ma G. Papadimitriou C. Gibson M. Corvelli M. Elisseeff J. (2018). Extracellular matrix particle-glycosaminoglycan composite hydrogels for regenerative medicine applications. J. Biomed. Mater. Res. Part A 106, 147–159. 10.1002/jbm.a.36218 28879659

[B5] Beachley V. Z. Wolf M. T. Sadtler K. Manda S. S. Jacobs H. Blatchley M. R.. (2015). Tissue matrix arrays for high-throughput screening and systems analysis of cell function. Nat. Methods 12, 1197–1204. 10.1038/nmeth.3619 26480475 PMC4666781

[B6] Beck E. C. Barragan M. Libeer T. B. Kieweg S. L. Converse G. L. Hopkins R. A.. (2016). Chondroinduction from naturally derived cartilage matrix: a comparison between devitalized and decellularized cartilage encapsulated in hydrogel pastes. Tissue Eng. Part A 22, 665–679. 10.1089/ten.tea.2015.0546 27001140 PMC4840832

[B7] Bellows C. G. Aubin J. E. Heersche J. N. (1991). Initiation and progression of mineralization of bone nodules formed *in vitro*: the role of alkaline phosphatase and organic phosphate. Bone Miner. 14, 27–40. 10.1016/0169-6009(91)90100-E 1868267

[B8] Beringer L. T. Li S. Kallick E. J. Shields K. J. Faight E. M. Cartieri F. (2016). Promoting adipogenesis using a collagen VI–heparin sulfate coating: applications in tissue engineering for wound healing. Ind. Eng. Chem. Res. 55, 12687–12692. 10.1021/acs.iecr.6b03567

[B9] Bitar M. Brown R. A. Salih V. Kidane A. G. Knowles J. C. Nazhat S. N. (2008). Effect of cell density on osteoblastic differentiation and matrix degradation of biomimetic dense collagen scaffolds. Biomacromolecules 9, 129–135. 10.1021/bm701112w 18095652

[B10] Boskey A. L. Posner A. S. (1984). Bone structure, composition, and mineralization. Orthop. Clin. North Am. 15, 597–612. 6387574

[B11] Brown C. F. C. Yan J. Han T. T. Y. Marecak D. M. Amsden B. G. Flynn L. E. (2015). Effect of decellularized adipose tissue particle size and cell density on adipose-derived stem cell proliferation and adipogenic differentiation in composite methacrylated chondroitin sulphate hydrogels. Biomed. Mater. 10:045010. 10.1088/1748-6041/10/4/045010 26225549

[B12] Bryant S. J. Davis-Arehart K. A. Luo N. Shoemaker R. K. ArthurKristi J. A. Anseth K. S. (2004). Synthesis and characterization of photopolymerized multifunctional hydrogels: water-soluble poly(vinyl alcohol) and chondroitin sulfate macromers for chondrocyte encapsulation. Macromolecules 37, 6726–6733. 10.1021/ma0499324

[B13] Cebotari S. Tudorache I. Jaekel T. Hilfiker A. Dorfman S. Ternes W.. (2010). Detergent decellularization of heart valves for tissue engineering: toxicological effects of residual detergents on human endothelial cells. Artif. Organs 34, 206–210. 10.1111/j.1525-1594.2009.00796.x 20447045

[B14] Chen Q. Shou P. Zheng C. Jiang M. Cao G. Yang Q. (2016a). Fate decision of mesenchymal stem cells: adipocytes or osteoblasts? Cell Death Differ. 23, 1128–1139. 10.1038/cdd.2015.168 26868907 PMC4946886

[B15] Chen S. Zhang M. Shao X. Wang X. Zhang L. Xu P. (2015). A laminin mimetic peptide SIKVAV-conjugated chitosan hydrogel promoting wound healing by enhancing angiogenesis, re-epithelialization and collagen deposition. J. Mater. Chem. B 3, 6798–6804. 10.1039/C5TB00842E 32262473

[B16] Chen Y. S. Chen Y. Y. Hsueh Y. S. Tai H. C. Lin F. H. (2016b). Modifying alginate with early embryonic extracellular matrix, laminin, and hyaluronic acid for adipose tissue engineering. J. Biomed. Mater. Res. Part A 104, 669–677. 10.1002/jbm.a.35606 26514819

[B17] Cheung H. Han T. T. Y. Marecak D. M. Watkins J. F. Amsden B. G. Flynn L. E. (2014). Biomaterials composite hydrogel scaffolds incorporating decellularized adipose tissue for soft tissue engineering with adipose-derived stem cells. Biomaterials 35, 1914–1923. 10.1016/j.biomaterials.2013.11.067 24331712

[B18] Chung C. H. Golub E. E. Forbes E. Tokuoka T. Shapiro I. M. (1992). Mechanism of action of beta-glycerophosphate on bone cell mineralization. Calcif. Tissue Int. 51, 305–311. 10.1007/BF00334492 1422975

[B19] Cortiella J. Niles J. Cantu A. Brettler A. Pham A. Vargas G.. (2010). Influence of ccellular natural lung matrix on murine embryonic stem cell differentiation. Tissue Eng. Part A 16, 2565–2580. 10.1089/ten.tea.2009.0730 20408765

[B20] de Girolamo L. Sartori M. F. Albisetti W. Brini A. T. (2007). Osteogenic differentiation of human adipose-derived stem cells: comparison of two different inductive media. J. Tissue Eng. Regen. Med. 1, 154–157. 10.1002/term.12 18038404

[B21] DeQuach J. A. Yuan S. H. Goldstein L. S. B. Christman K. L. (2011). Decellularized porcine brain matrix for cell culture and tissue engineering scaffolds. Tissue Eng. Part A 17, 2583–2592. 10.1089/ten.tea.2010.0724 21883047 PMC3204197

[B22] Figueiredo M. Henriques J. Martins G. Guerra F. Judas F. Figueiredo H. (2010). Physicochemical characterization of biomaterials commonly used in dentistry as bone substitutes-comparison with human bone. J. Biomed. Mater. Res. Part B Appl. Biomater. 92B, 409–419. 10.1002/jbm.b.31529 19904820

[B23] Flynn L. Semple J. L. Woodhouse K. A. (2006). Decellularized placental matrices for adipose tissue engineering. J. Biomed. Mater. Res. Part A 79A, 359–369. 10.1002/jbm.a.30762 16883587

[B24] Flynn L. E. (2010). The use of decellularized adipose tissue to provide an inductive microenvironment for the adipogenic differentiation of human adipose-derived stem cells. Biomaterials 31, 4715–4724. 10.1016/j.biomaterials.2010.02.046 20304481

[B25] French K. M. Boopathy A. V. DeQuach J. A. Chingozha L. Lu H. Christman K. L.. (2012). A naturally derived cardiac extracellular matrix enhances cardiac progenitor cell behavior *in vitro*. Acta Biomater. 8, 4357–4364. 10.1016/j.actbio.2012.07.033 22842035 PMC3488121

[B26] Gardin C. Ricci S. Ferroni L. Guazzo R. Sbricoli L. De Benedictis G.. (2015). Decellularization and delipidation protocols of bovine bone and pericardium for bone grafting and guided bone regeneration procedures. PLoS ONE 10, 1–26. 10.1371/journal.pone.0132344 26191793 PMC4507977

[B27] Han T. T. Y. Toutounji S. Amsden B. G. Flynn L. E. (2015). Adipose-derived stromal cells mediate *in vivo* adipogenesis, angiogenesis and inflammation in decellularized adipose tissue bioscaffolds. Biomaterials 72, 125–137. 10.1016/j.biomaterials.2015.08.053 26360790

[B28] Hayami J. W. S. Waldman S. D. Amsden B. G. (2015). Photo-cross-linked methacrylated polysaccharide solution blends with high chondrocyte viability, minimal swelling, and moduli similar to load bearing soft tissues. Eur. Polym. J. 72, 1–11. 10.1016/j.eurpolymj.2015.01.038

[B29] He X. Ma J. Jabbari E. (2008). Effect of grafting RGD and BMP-2 protein-derived peptides to a hydrogel substrate on osteogenic differentiation of marrow stromal cells. Langmuir 24, 12508–12516. 10.1021/la802447v 18837524

[B30] Hoshiba T. Lu H. Kawazoe N. Chen G. (2010). Decellularized matrices for tissue engineering. Expert Opin. Biol. Ther. 10, 1717–1728. 10.1517/14712598.2010.534079 21058932

[B31] Hsueh Y.S. Chen Y.S. Tai H.C. Mestak O. Chao S.C. Chen Y.Y.. (2017). Laminin-alginate beads as preadipocyte carriers to enhance adipogenesis *in vitro* and *in vivo*. Tissue Eng. Part A 23, 185–194. 10.1089/ten.tea.2016.0290 27814669

[B32] Huang W. Yang S. Shao J. Li Y. P. (2007). Signaling and transcriptional regulation in osteoblast commitment and differentiation. Front. Biosci. 12, 3068–3092. 10.2741/2296 17485283 PMC3571113

[B33] Hung B. P. Naved B. A. Nyberg E. L. Dias M. Holmes C. A. Elisseeff J. H.. (2016). Three-dimensional printing of bone extracellular matrix for craniofacial regeneration. ACS Biomater. Sci. Eng. 2, 1806–1816. 10.1021/acsbiomaterials.6b00101 27942578 PMC5142751

[B34] Junqueira L. C. Bignolas G. Brentani R. R. (1979). Picrosirius staining plus polarization microscopy, a specific method for collagen detection in tissue sections. Histochem. J. 11, 447–55. 10.1007/BF01002772 91593

[B35] Kasten A. Naser T. Brüllhoff K. Fiedler J. Müller P. Möller M.. (2014). Guidance of mesenchymal stem cells on fibronectin structured hydrogel films. PLoS ONE 9:e109411. 10.1371/journal.pone.0109411 25329487 PMC4198140

[B36] Kawaguchi N. Toriyama K. Nicodemou-Lena E. Inou K. Torii S. Kitagawa Y. (1999). Reconstituted basement membrane potentiates *in vivo* adipogenesis of 3T3-F442A cells. Cytotechnology 31, 215–220. 10.1023/A:1008198731341 19003144 PMC3449540

[B37] Lee D. J. Diachina S. Lee Y. T. Zhao L. Zou R. Tang N.. (2016). Decellularized bone matrix grafts for calvaria regeneration. J. Tissue Eng. 7:204173141668030. 10.1177/2041731416680306 28228929 PMC5308431

[B38] McBeath R. Pirone D. M. Nelson C. M. Bhadriraju K. Chen C. S. (2004). Cell shape, cytoskeletal tension, and RhoA regulate stem cell lineage commitment. Dev. Cell 6, 483–495. 10.1016/S1534-5807(04)00075-9 15068789

[B39] Morissette Martin P. Grant A. Hamilton D. W. Flynn L. E. (2019). Matrix composition in 3-D collagenous bioscaffolds modulates the survival and angiogenic phenotype of human chronic wound dermal fibroblasts. Acta Biomater. 83, 199–210. 10.1016/j.actbio.2018.10.042 30385224

[B40] Nakayama K. H. Lee C. C. I. Batchelder C., A. Tarantal A. F. (2013). Tissue specificity of decellularized rhesus monkey kidney and lung scaffolds. PLoS ONE 8:e64134. 10.1371/journal.pone.0064134 23717553 PMC3661477

[B41] Nguyen B. N. B. Moriarty R. A. Kamalitdinov T. Etheridge J. M. Fisher J. P. (2017). Collagen hydrogel scaffold promotes mesenchymal stem cell and endothelial cell coculture for bone tissue engineering. J. Biomed. Mater. Res. A 105, 1123–1131. 10.1002/jbm.a.36008 28093887 PMC5328802

[B42] Nicodemus G. D. Bryant S. J. (2008). Cell encapsulation in biodegradable hydrogels for tissue engineering applications. Tissue Eng. Part B Rev. 14, 149–165. 10.1089/ten.teb.2007.0332 18498217 PMC2962861

[B43] Ondeck M. G. Engler A. J. (2016). Mechanical characterization of a dynamic and tunable methacrylated hyaluronic acid hydrogel. J. Biomech. Eng. 138:021003. 10.1115/1.4032429 26746491 PMC4844086

[B44] Ornell K. J. Lozada D. Phan N. V. Coburn J. M. (2019). Controlling methacryloyl substitution of chondroitin sulfate: injectable hydrogels with tunable long-term drug release profiles. J. Mater. Chem. B 7, 2151–2161. 10.1039/C8TB03020K 32073574

[B45] Pacaccio D. J. Stern S. F. (2005). Demineralized bone matrix: basic science and clinical applications. Clin. Podiatr. Med. Surg. 22, 599–606. 10.1016/j.cpm.2005.07.001 16213382

[B46] Paduano F. Marrelli M. Alom N. Amer M. White L. J. Shakesheff K. M.. (2017). Decellularized bone extracellular matrix and human dental pulp stem cells as a construct for bone regeneration. J. Biomater. Sci. Polym. Ed. 28, 730–748. 10.1080/09205063.2017.1301770 28285576

[B47] Park J. Park J. Nahm S. S. Choi I. Kim J. (2013). Identification of anti-adipogenic proteins in adult bovine serum suppressing 3T3-L1 preadipocyte differentiation. BMB Rep. 46, 582–587. 10.5483/BMBRep.2013.46.12.082 24195790 PMC4133865

[B48] Peterson B. Whang P. G. Iglesias R. Wang J. C. Lieberman J. R. (2004). Osteoinductivity of commercially available demineralized bone matrix. Preparations in a spine fusion model. J. Bone Joint Surg. Am. 86, 2243–2250. 10.2106/00004623-200410000-00016 15466734

[B49] Ravindran S. Huang C. C. Gajendrareddy P. Narayanan R. (2015). Biomimetically enhanced demineralized bone matrix for bone regenerative applications. Front. Physiol. 6:292. 10.3389/fphys.2015.00292 26557093 PMC4617051

[B50] Schäck L. M. Noack S. Winkler R. Wißmann G. Behrens P. Wellmann M.. (2013). The phosphate source influences gene expression and quality of mineralization during *in vitro* osteogenic differentiation of human mesenchymal stem cells. PLoS ONE 8:e65943. 10.1371/journal.pone.0065943 23823126 PMC3688813

[B51] Scott J. E. (1995). Extracellular matrix, supramolecular organisation and shape. J. Anat. 187, 259–269. 7591990 PMC1167422

[B52] Shridhar A. Gillies E. Amsden B. G. Flynn L. E. (2017). Composite bioscaffolds incorporating decellularized ECM as a cell-instructive component within hydrogels as *in vitro* models and cell delivery systems. Methods Mol. Biol. 1577, 183–208. 10.1007/7651_2017_36 28493212

[B53] Slater M. Patava J. Mason R. S. (2009). Role of chondroitin sulfate glycosaminoglycans in mineralizing osteoblast-like cells: effects of hormonal manipulation. J. Bone Miner. Res. 9, 161–169. 10.1002/jbmr.5650090205 8140929

[B54] Stern M. M. Myers R. L. Hammam N. Stern K. A. Eberli D. Kritchevsky S. B.. (2009). The influence of extracellular matrix derived from skeletal muscle tissue on the proliferation and differentiation of myogenic progenitor cells *ex vivo*. Biomaterials 30, 2393–2399. 10.1016/j.biomaterials.2008.12.069 19168212 PMC9850446

[B55] Strioga M. Viswanathan S. Darinskas A. Slaby O. Michalek J. (2012). Same or not the same? comparison of adipose tissue-derived versus bone marrow-derived mesenchymal stem and stromal cells. Stem Cells Dev. 21, 2724–2752. 10.1089/scd.2011.0722 22468918

[B56] Turner A. E. B. Flynn L. E. (2012). Design and characterization of tissue-specific extracellular matrix-derived microcarriers. Tissue Eng. Part C Methods 18, 186–197. 10.1089/ten.tec.2011.0246 21981618

[B57] Uriel S. Huang J. J. Moya M. L. Francis M. E. Wang R. Chang S.. (2008). The role of adipose protein derived hydrogels in adipogenesis. Biomaterials 29, 3712–3719. 10.1016/j.biomaterials.2008.05.028 18571717

[B58] Visser J. Levett P. A. te Moller N. C. R. Besems J. Boere K. W. M. van Rijen M. H. P.. (2015). Crosslinkable hydrogels derived from cartilage, meniscus, and tendon tissue. Tissue Eng. Part A 21, 1195–1206. 10.1089/ten.tea.2014.0362 25557049 PMC4394887

[B59] Williams C. Sullivan K. Black L. D. (2015). Partially digested adult cardiac extracellular matrix promotes cardiomyocyte proliferation *in vitro*. Adv. Healthc. Mater. 4, 1545–1554. 10.1002/adhm.201500035 25988681 PMC4504755

[B60] Xue R. Yi-Shuan Li J. Yeh Y. Yang L. Chien S. (2013). Effects of matrix elasticity and cell density on human mesenchymal stem cells differentiation. J. Orthop. Res. 31, 1360–1365. 10.1002/jor.22374 23606500

[B61] Yahia L. H. Drouin G. (1990). Study of the hysteresis phenomenon in canine anterior cruciate ligaments. J. Biomed. Eng. 12, 57–62. 10.1016/0141-5425(90)90116-5 2296171

[B62] Yu C. Bianco J. Brown C. Fuetterer L. Watkins J. F. Samani A.. (2013). Porous decellularized adipose tissue foams for soft tissue regeneration. Biomaterials 34, 3290–3302. 10.1016/j.biomaterials.2013.01.056 23384795

[B63] Yu C. Kornmuller A. Brown C. Hoare T. Flynn L. E. (2017). Decellularized adipose tissue microcarriers as a dynamic culture platform for human adipose-derived stem/stromal cell expansion. Biomaterials 120, 66–80. 10.1016/j.biomaterials.2016.12.017 28038353

[B64] Zwick R. K. Guerrero-Juarez C. F. Horsley V. Plikus M. V. (2018). Anatomical, physiological, and functional diversity of adipose tissue. Cell Metab. 27, 68–83. 10.1016/j.cmet.2017.12.002 29320711 PMC6050204

