# Supplementary material for: Investigating the Effects of Tissue-Specific Extracellular Matrix on the Adipogenic and Osteogenic Differentiation of Human Adipose-Derived Stromal Cells Within Composite Hydrogel Scaffolds
Source: Front Bioeng Biotechnol. 2019 Dec 11;7:402. doi: 10.3389/fbioe.2019.00402 (PMC6917659; doi:10.3389/fbioe.2019.00402)
Supplement: Supplementary file 1 [file Table_1.docx]

Supplementary Material


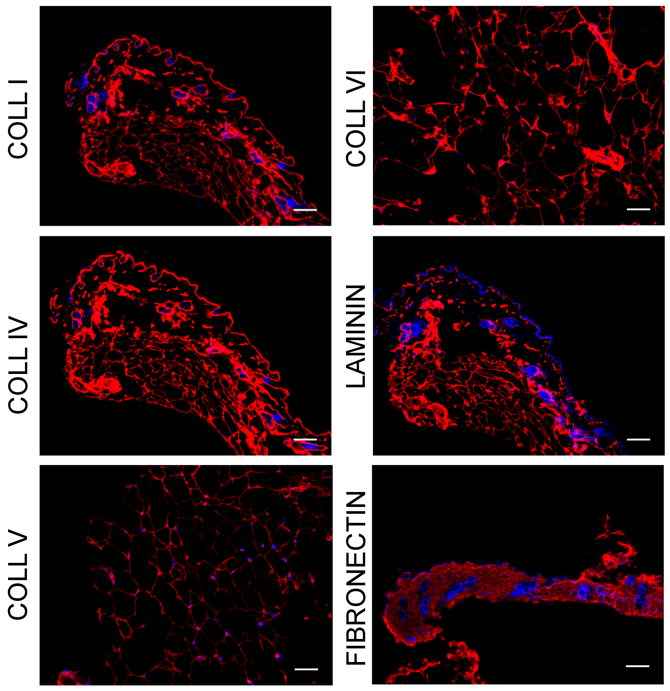


**Supplementary Figure S1:** **Representative images of positive tissue controls for immunohistochemical staining.** Mouse skin was used as a tissue type control for collagen type I, IV, laminin and fibronectin, while human adipose tissue was used as a tissue type control for collagen type V and VI. Scale:100 μm.


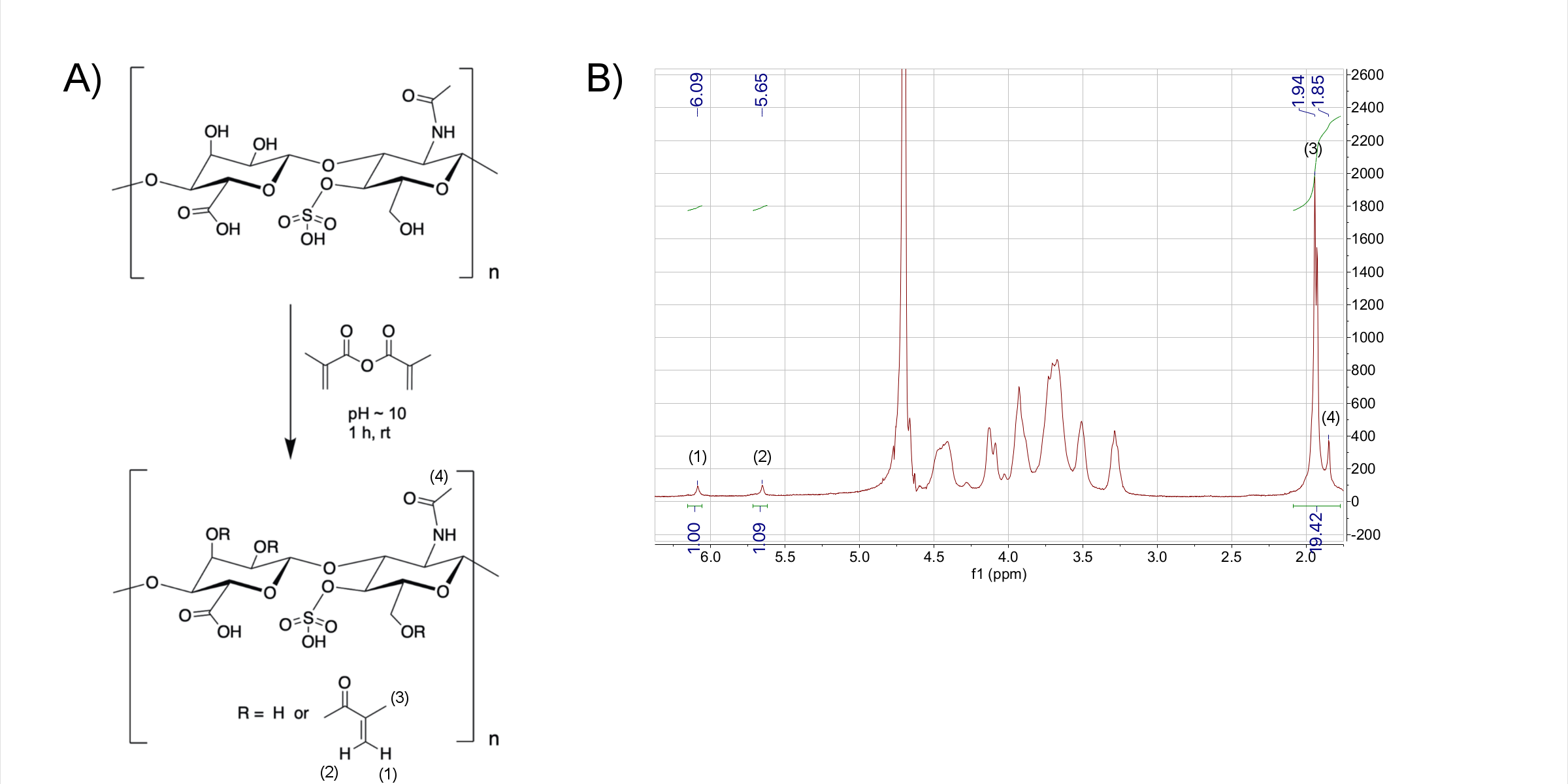


Supplementary Figure S2: (A) Reaction scheme for the methacrylation of chondroitin sulphate and (B) ^1^H NMR spectrum (400 MHz, D_2_O) of the resulting methacrylated chondroitin sulphate. The peaks at 5.65, 6.09 and 1.85 correspond to the protons from the newly grafted methacrylate group, while the peak at 1.85 corresponds to the protons from the methyl group of the N-acetyl residues in native CS.


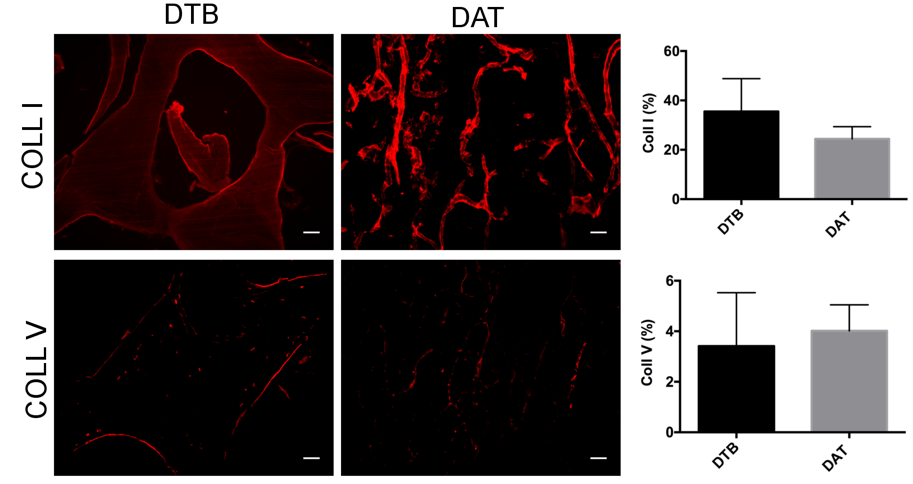


Supplementary Figure S3: Immunohistochemical staining and semi-quantitative comparative analyses of the DTB and DAT. Analyses suggested no statistical difference in the levels of collagen type I and V between the groups. (n=3, N=2 tissue donors, 4-5 images per sample) Scale: 100 μm.


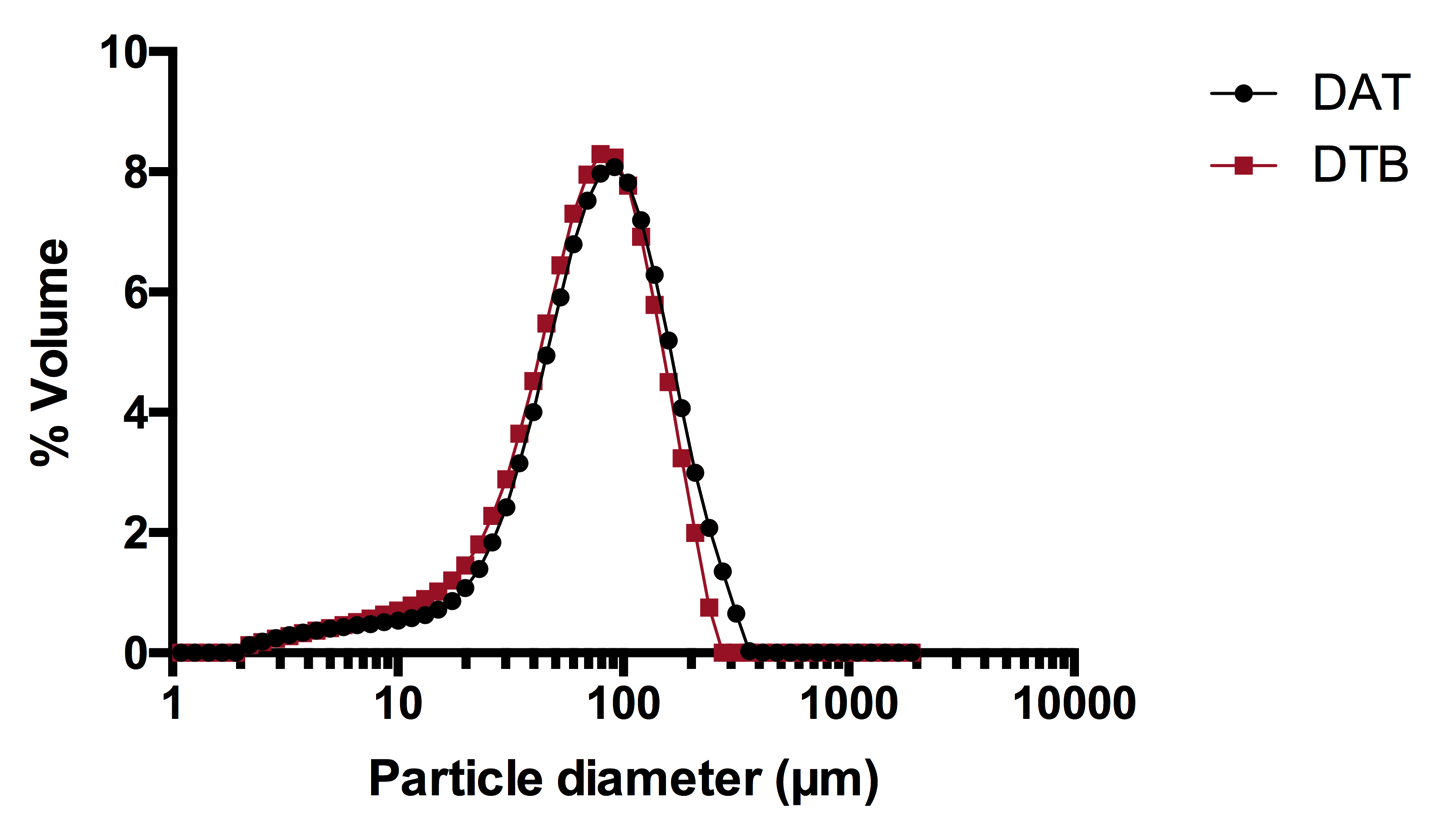


Supplementary Figure S4: Particle size distribution of ECM particles generated after cryo-milling and sieving between 45 μm and 125 μm stainless steel mesh filters. ECM particles were pooled from multiple donors and the same batch was used for all studies. Data indicated that a distribution of particle diameters between 2 μm and 200 μm was obtained, with the majority of the particles lying between the size range of sieves used (45 μm and 125 μm). Additionally, there was no significant difference in particle size between the DAT and the DTB.


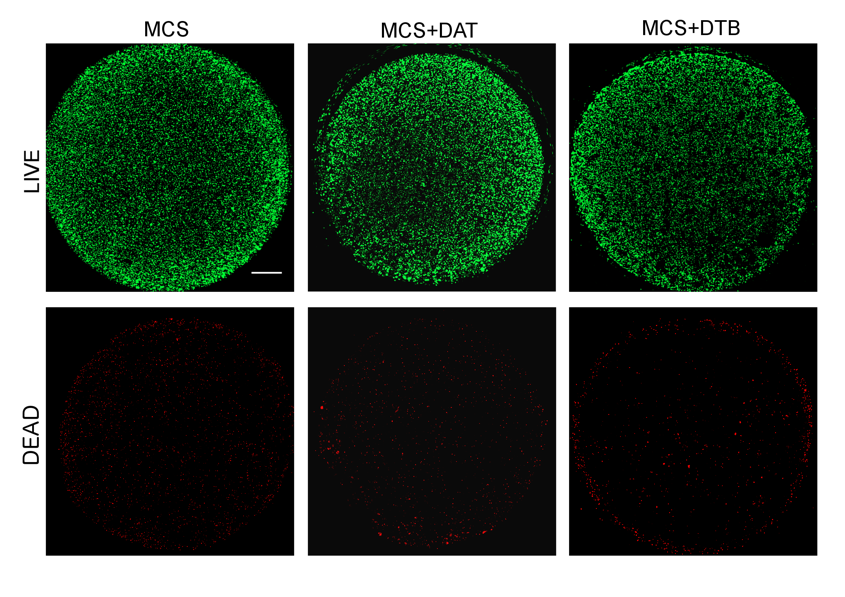


Supplementary Figure S5: Representative confocal microscopy images of LIVE/DEAD®-stained ASCs encapsulated within an MCS, MCS+DAT and MCS+DTB hydrogel at 7 d. Live cells (calcein-AM^+^) are shown at the top in green and dead cells (Ethidium homodimer-1^+^) at the bottom in red. Scale:1 mm

**Supplementary Table S1: Summary of cell donor information for all *in vitro* studies.**

| **Study** | | **Donor information** |
| --- | --- | --- |
| **Viability** | **LIVE/DEAD®** | **Donor 1:** 62 yr, BMI=30.1 **Donor 2:** 37 yr,BMI=29 **Donor 3:** 39 yr, BMI=30 |
| **Adipogenic Differentiation** | **GPDH/BODIPY** | **Donor 1:** 59 yr, BMI=26 **Donor 2:** 59 yr, BMI=29 **Donor 3:** 58 yr, BMI=30.4 |
| **Osteogenic Differentiation** | **ALP** | **Donor 1:** 66 yr, BMI=25.8 **Donor 2:** 58 yr, BMI=25.6 **Donor 3:** 35 yr, BMI=21.8 |
|  | **Matrix mineralization** | **Donor 1:** 66 yr, BMI=22.2 **Donor 2:** 66 yr, BMI=22.8 |
